# Laparoscopic complete mesocolic excision with D3 lymph node dissection for right colon cancer in elderly patients

**DOI:** 10.1038/s41598-020-69617-4

**Published:** 2020-07-28

**Authors:** Duk Yeon Hwang, Gyeo Ra Lee, Ji Hoon Kim, Yoon Suk Lee

**Affiliations:** 1 Department of Surgery, Incheon St. Mary’s Hospital, College of Medicine, The Catholic University of Korea, 56 Dongsu-ro, Bupyeong-gu, Incheon, Republic of Korea; 2 Department of Surgery, Seoul St. Mary’s Hospital, College of Medicine, The Catholic University of Korea, 222 Banpodae-ro, Seocho-gu, Seoul, Republic of Korea

**Keywords:** Cancer, Gastrointestinal cancer, Colorectal cancer, Colon cancer, Surgical oncology

## Abstract

Complete mesocolic excision (CME) with D3 lymph node dissection is considered an oncological surgery for right colon cancer. However, there is still controversy for extensive oncological surgery in elderly patients. The aim of this study is to evaluate the safety and oncological outcomes of laparoscopic CME with D3 lymph node dissection for right colon cancer in elderly patients. Patients who underwent laparoscopic right colectomy, from 2004 to 2014, were divided into Groups A (age ≥ 70 years, n = 80) or B (age < 70 years, n = 127). Short and long-term outcomes were analysed. Basic demographics and short-term surgical outcomes were similar between groups. Among pathological outcomes, the mean number of harvested lymph nodes was significantly less in Group A. Adjuvant chemotherapy refusal rate was significantly higher in Group A. Overall and recurrence-free survival were similar between groups. We found laparoscopic CME with D3 lymph node dissection is a safe and feasible surgical option for right colon cancer in elderly patients.

## Introduction

The increase in life expectancy is a distinguishing feature of modern society, resulting in higher demands for health and welfare services. Colorectal cancer is one of the most common malignancies not only in Western countries but also in north-east Asia. According to the 2016 Korean cancer statistics, colorectal cancer is the second most common cancer and the third leading cause of cancer-related death in the Republic of Korea^1^. Therefore, an increased proportion of elderly patients suffering from colorectal cancer will be candidates for surgery.

Since its introduction in 1991, laparoscopic colorectal surgery has become a popular treatment of choice. Since reduced surgical invasiveness may result in fewer postoperative complications and faster postoperative recovery, laparoscopic surgery is considered a good option in elderly patients. Most controlled studies assessing the benefits of laparoscopic colectomy in elderly patients have reported advantages of laparoscopic colectomy over open colectomy^2–7^.

Complete mesocolic excision (CME) with D3 lymph node dissection is considered an oncological surgical option for colon cancer. However, there is still a controversy about extensive radical lymph node dissection in elderly patients with colon cancer. Many have suggested that the elderly may have physiologic or anatomic differences compared to the younger population; therefore, there would be fewer lymph nodes in the resected mesentery of the elderly^8^. Several surgeons are less aggressive in lymph node dissection in the elderly based on the belief that extensive lymph node dissection may result in increased morbidity without benefit in a patient with a limited lifespan^9^. However, there are few studies which evaluate safety and oncological impact of extensive lymph node dissection in elderly patients. The aim of this study is to evaluate the short- and long-term outcomes of laparoscopic CME with D3 lymph node dissection of right colon cancer in elderly patients.

## Materials and methods

### Study design

A total 207 patients who had undergone laparoscopic CME with D3 lymph node dissection from 2004 to 2014 for right colon cancer at Incheon St. Mary’s Hospital, The Catholic University of Korea, were enrolled. Patients were divided into two groups: Group A, which consisted of elderly patients (age ≥ 70 years, n = 80), or Group B, which consisted of younger patients (age < 70 years, n = 127). Demographic and operational data, pathologic outcomes, postoperative short-term clinical outcomes, overall survival, and recurrence-free survival were analysed between the two groups. Patients in whom R1, R2 resection or synchronous multiple cancers, combined organ resection, and stage IV colon cancer were excluded from this study.

### Surgical procedure and D3 Lymph node dissection

**Figure 1:**
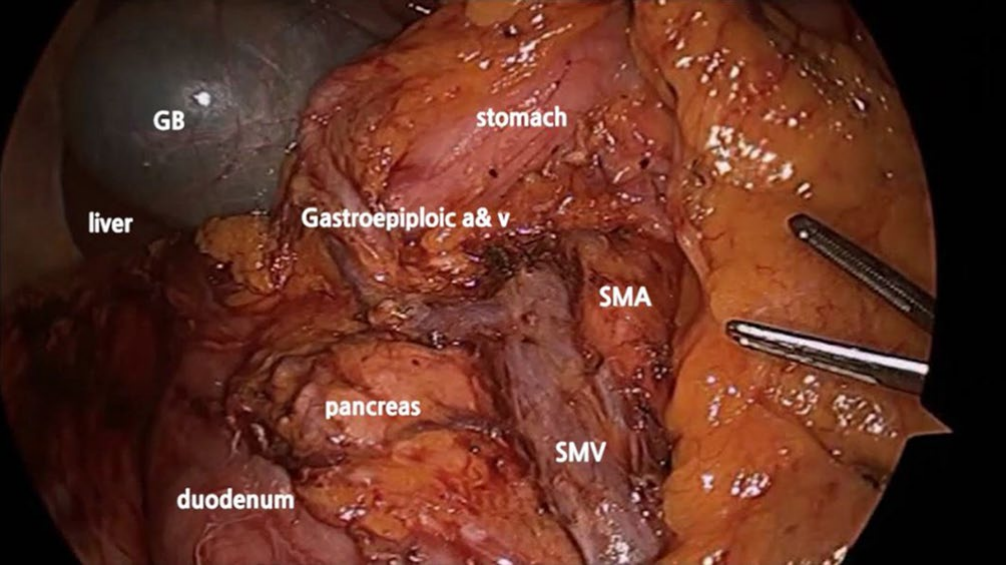
Complete mesocolic excision (CME) with D3 lymph node dissection.

D3 lymph node dissection was defined as removal of main lymph nodes at the root of the feeding vessels (ileocolic vessels and the right branch of the middle colic artery or middle colic artery), followed by ligation of vessels at the origin site (Fig. 1).

### Postoperative complications

Intraoperative major complications included massive bleeding, which required transfusion, and organ injury, which required surgical treatment. The postoperative surgical complications in accordance with Clavien-Dindo classification $\ge$ 2 were investigated. These included surgical site infection, ileus, anastomotic leakage, bleeding, organ dysfunction, and sepsis.

### Statistical analyses

Continuous variables such as age, operation time, amount of bleeding, and number of harvested lymph nodes are presented as mean ± standard deviation. Categorical variables, such as sex and surgical complication ratio, are expressed as frequencies or ratios. Student’s t-test was used to compare continuous variables, whereas the categorical variables were compared using the Chi-square test. Among categorical variables, the American Society of Anesthesiologists (ASA) score, intraoperative complication ratio and conversion ratio, and mortality ratios were compared using Fisher’s exact test, as appropriate. Survival was analysed using the Kaplan–Meier method, and the curves were compared using the log-rank test. A *P* value of $\le 0.05$ was considered significant.

All methods were performed in accordance with the relevant guidelines and regulations, and this study was approved by the Institutional Review Board of Incheon St. Mary’s Hospital (OC18RESI0020), which also waived the requirement for informed consent due to the retrospective nature of the study.

## Results

The demographic features of Groups A and B are shown in Table 1. The mean age of Groups A and B was 76.3 years and 57.9 years, respectively (*P* = 0.000). No significant differences were observed between the two groups in terms of sex, body mass index, and ASA physical status score. Table 2 shows no significant differences between the two groups regarding operation details. Major intraoperative complications such as duodenal injury and bleeding due to major vessel injury occurred in 3 cases in Group A and 4 cases in Group B. The open conversion ratio was 2/80 (2.5%) and 3/127 (2.4%) in Groups A and B, respectively. The reasons for open conversion were large tumour size, congenital vascular anomaly, and bleeding due to major vessel injury. There were no significant differences between the two groups regarding pathologic TNM stage. The number of mean harvested lymph nodes was less in Group A than in Group B (25.3 ± 8.2 vs 31.4 ± 16.7, *P* = 0.001). No pathologic differences were noted regarding proximal resection margin, distal resection margin, tumour size, lymphatic, vascular, or perineural invasion between the two groups (Table 3). The postoperative clinical outcomes between the two groups are shown in Table 4. Postoperative hospital stay, diet start day, and flatus passing day were not significantly different between the two groups. Clavien-Dindo $\ge$ 2 postoperative complications occurred in 13 cases in Group A and 16 cases in Group B. These complications included postoperative bleeding, anastomotic leakage, ileus, acute renal failure, and pancreatitis. A single occurrence of mortality was recorded in Group A due to pulmonary thromboembolism. Refusal rate for adjuvant chemotherapy was significantly higher in Group A than in Group B (12/44 vs 5/77, *P* = 0.002).

**Table 1:** Patient demographics.

	Group A	Group B	*p* value
Age (years)	76.3 ± 5.0	57.9 ± 9.4	0.000
Sex (M:F)	36:44	72:55	0.101
Body mass index (BMI)	23.9 ± 3.4	23.4 ± 3.6	0.398
ASA 1–2:ASA 3	73:7	123:4	0.111

*M* male, *F* female, *ASA* American Society of Anesthesiologists physical status score, *BMI* body mass index.

**Table 2:** Operation details.

	Group A	Group B	*p* value
Operation time (minutes)	147.8 ± 49.1	150.6 ± 58.5	0.722
Intraoperative blood loss (cc)	75.4 ± 87.4	77.0 ± 123.6	0.921
Intraoperative major complication	3/80	4/127	0.552
Major vessel injury	2	1	
Duodenum injury	1	3	
Open conversion	2/80	3/127	0.642

**Table 3:** Pathologic outcomes.

	Group A	Group B	*p* value
**Pathologic TNM stage**			0.094
Stage I	11/80	31/127	
Stage II	37/80	43/127	
Stage III	32/80	53/127	
Mean number of harvested LN	25.3 ± 8.2	31.4 ± 16.7	0.001
PRM (cm)	19.5 ± 8.1	19.6 ± 7.8	0.911
DRM (cm)	14.9 ± 6.1	14.2 ± 4.9	0.328
Tumor size (cm^2^)	5.6 ± 2.2	5.1 ± 3.0	0.128
Lymphatic invasion	35/80	52/127	0.773
Vascular invasion	6/80	14/127	0.403
Perineural invasion	32/80	35/127	0.062

*PRM* proximal resection margin, *DRM* distal resection margin, *LN* lymph nodes.

**Table 4:** Postoperative clinical outcomes.

	Group A	Group B	*p* value
Hospital stay (POD)	8.6 ± 3.6	9.2 ± 5.2	0.427
Diet start day	2.9 ± 1.5	2.9 ± 1.6	0.830
Flatus passing day	2.8 ± 1.0	2.7 ± 0.9	0.533
**Clavien-Dindo complication **$\ge$** 2**	13/80	16/127	0.461
Bleeding	2	2	
Pulmonary thromboembolism	1	0	
Anastomotic leakage	4	6	
Ileus	6	6	
Acute renal failure	0	1	
Pancreatitis	0	1	
Mortality case	1/80	0/127	0.386
Refuse rate of adjuvant chemotherapy	12/44	5/77	0.002

*POD* postoperative day, *SSI* surgical site infection.

**Figure 2:**
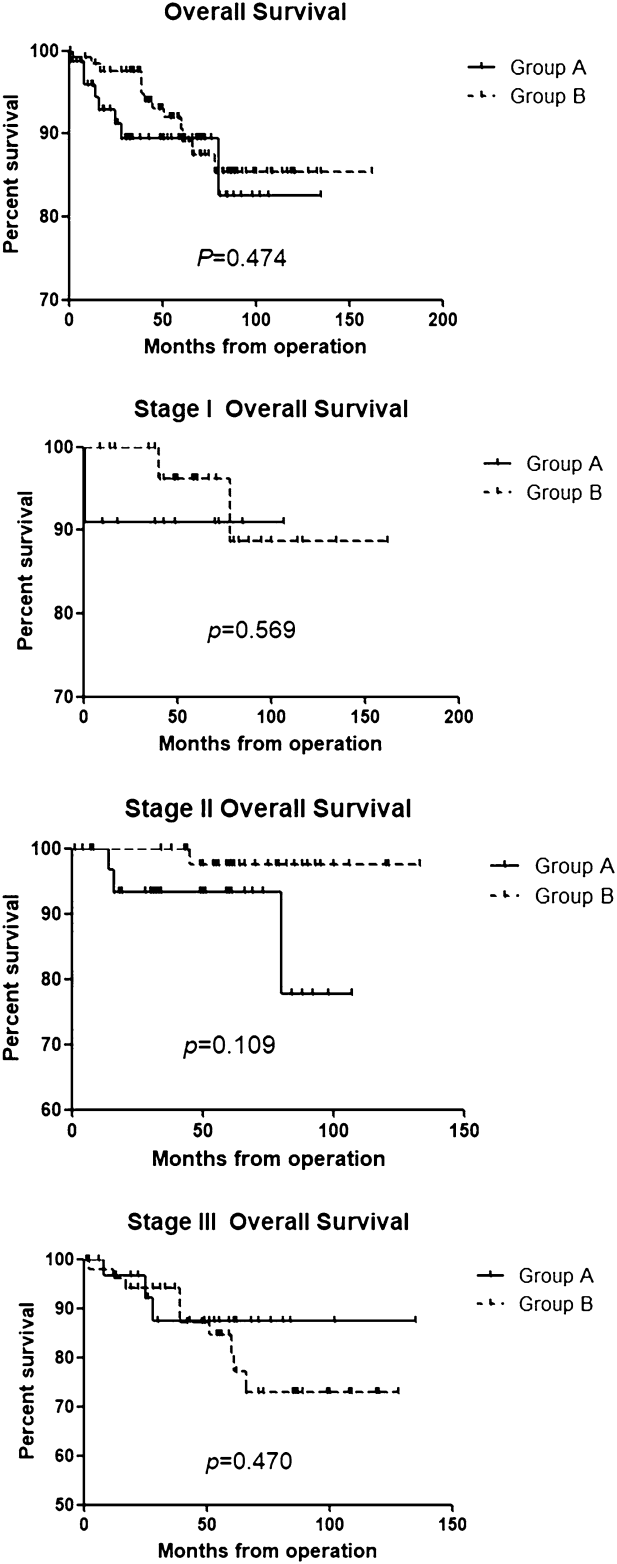
Overall survival.

**Figure 3:**
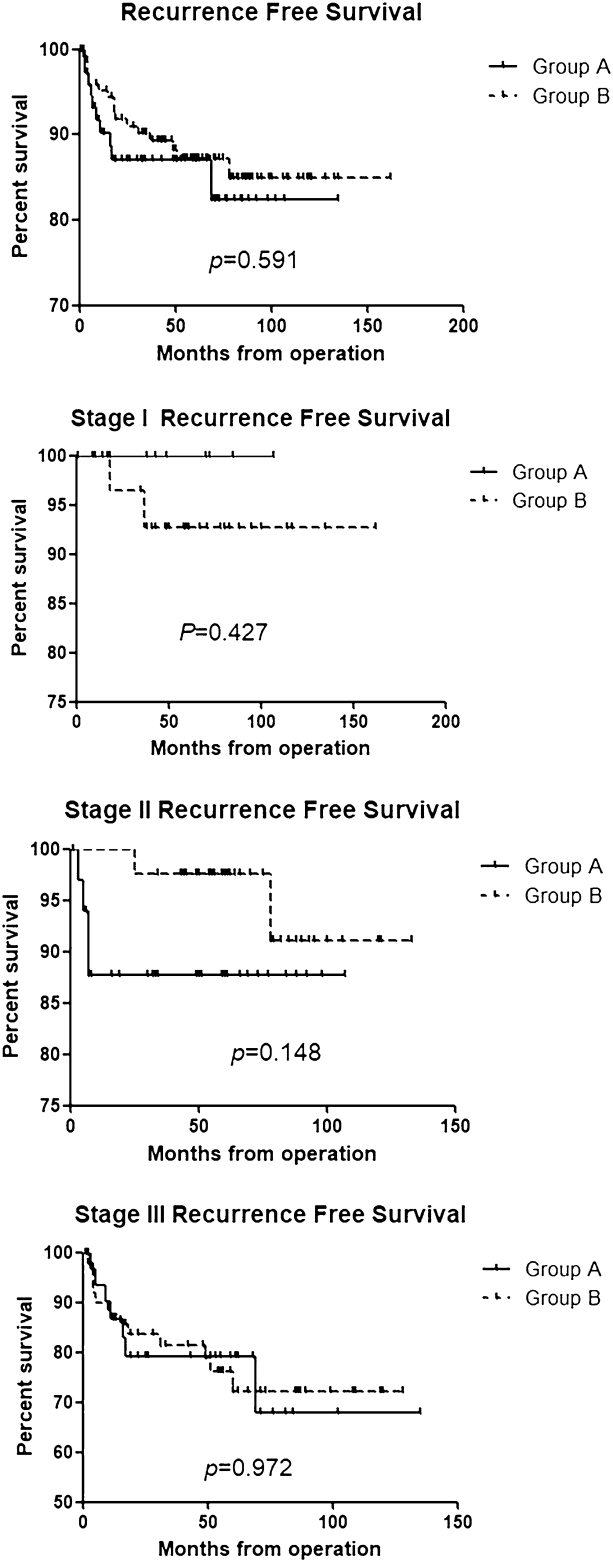
Recurrence-free survival.

Figures 2 and 3 show the survival analysis between the two groups. Overall survival and recurrence-free survival rates were not significantly different between the two groups according to each stage and all stage groups.

## Discussion

CME along with central vascular ligation of feeding vessel, commonly known as D3 lymph node dissection, was applied to colon cancer in a similar manner as total mesorectal excision for rectal cancer, in which embryologic tissue planes are resected along the entire enveloped mesocolon. This technique has been found to improve long-term survival and reduce local recurrence compared with previous performed procedures and is now being considered an extensive oncological surgical option for right colon cancer surgery^10,11^.

One of the most important prognostic factors in colorectal cancer is the nodal status. The presence of lymph node metastasis is a main determinant for adjuvant chemotherapy and a predictor of disease-free and overall survival^12–14^. Kotake et al. have reported that D3 lymph node dissection for pT3 and pT4 colon cancer is associated with a significant survival advantage from a large-scale database. Compared to D2 lymph node dissection, D3 lymph node dissection relatively reduces the risk of death by 18% in terms of the overall survival of patients with pT3 and pT4 colon cancer^15^.

Some previous studies have reported the effectiveness of D3 lymph node dissection, even in early stage colon cancer. Storli et al. reported that compared to the D2 approach, CME with D3 surgical management of colon cancer resulted in significant immediate improvement of 3-year survival for patients with TNM stage I-II tumours as assessed by their overall survival and disease-free survival^16^. This improvement was possibly due to the removal of the micrometastases and skip metastases in D3 lymph node dissection. Removal of micrometastases, which occur without obvious lymph node metastasis, influences the survival of patients with stage I and II colon cancer^17–20^.

There is debate regarding the extent of lymph node dissection for colon cancer between young and elderly patients. Egenvall et al. reported that elderly patients tend to be treated with less vigilance in surgical procedure of colon cancer performed than young patients. In particular, right colon cancer stage I-III is less often resected radically^21^. However, our study showed acceptable short-term clinical and long-term oncological outcomes of laparoscopic CME with D3 lymph node dissection in elderly patients compared to young patients. This study covered surgeries over an extended period, up to 10 years, and evolution of technique can affect the outcomes of study. Therefore, we conducted a subgroup analysis on perioperative complication rates, overall and recurrence-free survival between the two groups according to the 2004–2009 or 2010–2014 period. In this subgroup analysis, no difference was noted between the elderly and young patient groups. Specifically, in the 2004–2009 subgroup analysis, the *P* value of recurrence-free survival between the two groups was 0.326. The *P* value of overall survival between the two groups was 0.166. Perioperative complication ratio was 7/25 and 9/39 each, and the *P* value was 0.657. In the 2010–2014 subgroup analysis, the *P* value of recurrence-free survival between the two groups was 0.146 and the *P* value of overall survival between the two groups was 0.778. The perioperative complication ratio was 11/55 and 20/88, respectively, and the *P* value was 0.700. We think that it is important to offer the same standardised surgical resection procedure to all patients, regardless of age.

Laparoscopic CME with D3 lymph node dissection is technically demanding and carries the risk of several complications due to the complex anatomy related to the mesenteric planes and the vascular anatomical variations. Bertelsen et al. reported that CME is associated with more intraoperative organ injuries and severe non-surgical complications than conventional hemicolectomy^22^. However, several studies reported that laparoscopic D3 lymph node dissection for colon cancer is technically feasible and effective, with surgical results being optimised through a combination of surgical proficiency, institutional case load, and expertise^23–28^. In the present study, some intraoperative complications occurred, but most were managed appropriately during operation. The overall complication rate in this study was 16.3% in the elderly group and 12.6% in the younger group, but it was not significantly different.

Adjuvant chemotherapy improved oncological outcomes in locally advanced colorectal cancer and is usually recommended for locally advanced colorectal cancer. However, elderly patients are inclined to refuse adjuvant chemotherapy. Benson et al. reported that approximately 1/3 of elderly patients who had an indication for chemotherapy refused the treatment^29^. In this study, the refusal rate of adjuvant chemotherapy among candidates in the elderly group was 27.3% and was significantly higher than that in the younger group, which was 6.5%. Interestingly, no significant differences were however noted in the overall survival and recurrence-free survival between groups. We thought extensive lymph node dissection might have a favourable impact on oncological outcomes in elderly patients, even in patients who refused adjuvant chemotherapy.

This study has some limitations. First, because we performed D3 lymph node dissection in most of the patients, direct comparisons with D1 or D2 lymph node dissection in the same elderly group could not be made. Second, this study had a retrospective and single-centre design. Additional prospective studies with multiple centres are required to fully evaluate the safety and oncological impact of laparoscopic CME with D3 lymph node dissection in elderly patients with right colon cancer.

## Conclusion

Our study revealed acceptable short-term clinical outcomes and long-term oncologocical outcomes of laparoscopic CME with D3 lymph node dissection in elderly patients with right colon cancer, despite the high refusal rate of adjuvant chemotherapy. We recommend this procedure as a safe and optimal option for these patients.
